# Ramulus Mori (Sangzhi) Alkaloids Ameliorate Obesity-Linked Adipose Tissue Metabolism and Inflammation in Mice

**DOI:** 10.3390/nu14235050

**Published:** 2022-11-27

**Authors:** Qian-Wen Sun, Chun-Fang Lian, Yan-Min Chen, Jun Ye, Wei Chen, Yue Gao, Hong-Liang Wang, Li-Li Gao, Yu-Ling Liu, Yan-Fang Yang

**Affiliations:** 1 State Key Laboratory of Bioactive Substance and Function of Natural Medicines, Institute of Materia Medica, Chinese Academy of Medical Sciences & Peking Union Medical College, Beijing 100050, China; 2 Beijing Key Laboratory of Drug Delivery Technology and Novel Formulation, Institute of Materia Medica, Chinese Academy of Medical Sciences & Peking Union Medical College, Beijing 100050, China

**Keywords:** Ramulus Mori alkaloids, obesity, adipose tissue, lipid metabolism, adipose inflammation

## Abstract

Obesity has become a global epidemic disease as it is closely associated with a chronic low-grade inflammatory state that results in metabolic dysfunction. Ramulus Mori (Sangzhi) alkaloids (SZ-A) derived from *Morus alba* L. were licensed to treat type 2 diabetes (T2DM) in 2020. In this study, we explored the effect of SZ-A on adipose tissue metabolism and inflammation using an obesity model induced by a high-fat diet (HFD). C57BL/6J mice were fed high fat for 14 weeks and followed by SZ-A 400 mg/kg treatment via gavage for another six weeks, during which they were still given the high-fat diet. The results showed that SZ-A notably reduced body weight and serum levels of lipid metabolism-related factors, such as triglycerides (TG) and total cholesterol (TC); and inflammation-related factors, namely tumor necrosis factor alpha (TNFα), interleukin 6 (IL6), fibrinogen activator inhibitor-1 (PAI-1), angiopoietin-2 (Ang-2), and leptin (LEP), in the HFD-induced mice. SZ-A increased the protein and mRNA expression of lipid metabolism-related factors, including phosphorylated acetyl coenzyme A carboxylase (p-ACC), phosphorylated hormone-sensitive triglyceride lipase (p-HSL), adipose triglyceride lipase (ATGL), and peroxisome proliferator-activated receptor-alpha (PPARα), in adipose tissue. Immunohistochemistry results demonstrated that SZ-A significantly reduced the infiltration of pro-inflammatory M1-type macrophages in epididymal fat. The data also suggested that SZ-A down-regulates the transcriptional levels of inflammatory factors *Il6*, *Tnfα*, monocyte chemoattractant protein-1 (*Mcp1*), and *F4/80*, and up-regulates interleukin 4 (*Il4*), interleukin 10 (*Il10*), and interleukin 13 (*Il13*) in adipose tissue. Overall, the results indicate that SZ-A exhibits potential in regulating lipid metabolism and ameliorating obesity-linked adipose inflammation.

## 1. Introduction

Obesity has become a chronic and global epidemic disease because it is intimately related to a chronic low-grade inflammatory state and even exacerbates obesity-related diseases, particularly metabolic dysfunctions [1]. In patients with obesity, adipose tissue releases adipokines that trigger and maintain chronic low-grade inflammation and increase oxidative stress in the adipose tissue [2]. Controlling the progression of obesity and reducing weight have become important strategies in the treatment of numerous diseases, particularly metabolic diseases. Therefore, it is essential to develop safe and effective weight loss drugs to reduce the morbidity and mortality of various chronic diseases resulting from obesity.

White adipose tissue (WAT) is the primary organ for energy storage. WAT plays a crucial role in regulating lipid distribution and energy homeostasis in the body and is involved in the development of obesity. Adipose tissue is closely linked to metabolic health through the development of adipocyte hypertrophy and hyperplasia in which hypertrophic growth is a critical component in triggering adipose tissue dysfunction. Once energy intake exceeds consumption, the excess energy will be stored in the adipocytes in the form of triglycerides, leading to hypertrophic expansion. Acetyl-CoA carboxylase (ACC), an essential enzyme in the synthesis of fatty acids from acetyl-CoA, provides a substrate for triglyceride synthesis in adipocytes. Considering that phosphorylated acetyl-CoA carboxylase (p-ACC) loses catalytic capacity, promoting ACC phosphorylation could inhibit the fatty acid synthesis and reduce substrates required for triglyceride synthesis [3]. In contrast to lipogenesis, the triglycerides in adipocytes break down and release free glycerol and fatty acids, providing a substrate source for hepatic gluconeogenesis and oxidative catabolism. Adipocyte triglyceride lipase (ATGL) and phosphorylated hormone-sensitive lipase (p-HSL) are critical in initiating and catalyzing the triglyceride breakdown step wisely. Therefore, promoting the function of lipolytic enzymes may increase triglyceride catabolism in adipocytes and reduce lipid accumulation.

Adipose tissue is also an endocrine organ capable of secreting a wide range of cytokines that are closely linked to a range of obesity-induced chronic diseases [4]. Weight gain and obesity often result in the increased secretion of inflammatory factors and chemokines, including tumor necrosis factor alpha (TNFα), interleukin 6 (IL6), and monocyte chemoattractant protein-1 (MCP-1). These inflammatory factors and chemokines induce the accumulation of inflammatory cells such as pro-inflammatory M1-type macrophages in the adipose tissue [5]. They also trigger persistent chronic low-grade inflammation, particularly in hypertrophic abdominal adipose tissue. Reducing adipose inflammation in patients with obesity has been explored as a potential therapeutic strategy to prevent obesity-related metabolic and vascular complications [6,7].

Ramulus Mori (Sangzhi) alkaloid (SZ-A) tablets are derived from *Morus alba* L. (mulberry twig) and approved for the treatment of Type 2 diabetes (T2DM) in China (approval number Z20200002). In a multicenter, randomized, double-blind clinical trial, SZ-A exhibited excellent hypoglycaemic effect in the treatment of T2DM, with minimal gastrointestinal- side effects like flatulence during treatment [8]. Previous studies have reported that SZ-A could restore diabetic β-cells, improve insulin resistance, and modulate the gut microbiota and intestinal barrier integrity in KKAy mice with signs of obesity and diabetes [9,10]. Additionally, it has been reported that SZ-A could alleviate non-alcoholic fatty liver disease (NAFLD) in high-fat diet (HFD)-induced obese mice [11]. It was also demonstrated that SZ-A could inhibit body weight gain in HFD mice in a dose-dependent manner, with SZ-A at a dose of 400 mg/kg having the most significant effect. In addition, Gao et al. reported that SZ-A treatment showed anti-inflammatory effects in vitro by blocking p38 MAPK, ERK, and JNK signaling pathways in macrophages. Owing to the synergistic effect of SZ-A, its anti-inflammatory effect was higher than that of its components [12]. The analysis of tissue distribution results suggested a high distribution of SZ-A in adipose tissue after oral administration [13]. These results indicate the potential modulating effects of SZ-A on the adipose tissue. 

In this study, we aimed to investigate the effects of SZ-A on adipose tissue metabolism and inflammation development in an HFD-fed mouse model and assessed whether SZ-A can be potentially used in treating lipid metabolism-related diseases from these results.

## 2. Materials and Methods

### 2.1. Chemical and Reagents

SZ-A powder was friendly presented by Beijing Wehand-Bio Pharmaceutical Co., Ltd. (Beijing, China). β-actin(4970), ACC(3676), p-ACC(11818), HSL(18381), p-HSL(4139), ATGL(2439), F4/80(70076), and CD86(19589) primary antibodies were purchased from Cell Signaling Technology (Danvers, MA, USA). The PPARα primary antibody (ab126285) was obtained from Abcam (Cambridge, MA, USA). Horseradish peroxidase (HRP)-conjugated anti-rabbit secondary antibodies were obtained from Zhong Shan-Golden Bridge Biological Technology Co., Ltd. (Beijing, China).

### 2.2. Animals Experimental and Treatment

The Ethical review of experimental animal welfare was approved by the Beijing Laboratory Animal Research Center (ethical code: 2021050). The mice were housed at 24 °C with a 12 h light/dark cycle. Food and water were freely available. After 1 week of acclimatization to the environment, six-week-old male C57BL/6J mice were randomly divided into the following three groups: a normal control group (NC, *n* = 10), an HFD control group subjected to intragastric administration of saline (HFD, *n* = 10), and the SZ-A treatment group subjected to intragastric administration of SZ-A (SZ-A, *n* = 10). The NC was fed a maintenance feed, whereas the HFD groups were fed with 60 kcal% fat (cat. no. D12492; Research Diets Inc.) for 14 weeks. Subsequently, the mice were intragastrically administered either saline or SZ-A at a dose of 400 mg/kg/d for another 6 weeks. Mice in the HFD and SZ-A treatment groups were kept feeding an HFD until the end of the experiment. Weighed weekly during whole the experiment period. At the end of the experiment, the mice were fasted overnight from 17:00 the day before the autopsy until 9:00 the next day and then euthanized. Blood was collected by removing eyeballs, allowed to stand for 30 min at room temperature, and then centrifuged at 3000 rpm at 4 °C for 15 min to separate the serum. Adipose tissues, including epididymal adipose tissue (eWAT), inguinal adipose tissue (iWAT), and brown adipose tissue (BAT), were dissected, weighed, and kept in liquid nitrogen for 30 min. Finally, the tissues were stored at −80 °C.

### 2.3. Serum Biochemical Parameters

Serum triglycerides (TGs), total cholesterol (TC), low-density lipoprotein cholesterol (LDL-C), and high-density lipoprotein cholesterol (HDL-C) levels were determined using an automatic biochemistry analyzer (TOSHIBA TBA-40FR, Tokyo, Japan) with appropriate commercial assay kits from Biosino Biotechnology and Science, Inc. (Beijing, China). Serum levels of tumor necrosis factor alpha (TNFα), interleukin 6 (IL6), fibrinogen activator inhibitor-1 (PAI-1), angiopoietin-2 (Ang-2), and leptin (LEP) were determined using a mouse Multifactor test kit (R&D Systems, Minneapolis, MN, USA). Serum adiponectin (ADPN) levels were determined using a mouse adiponectin ELISA kit (Abcam, Cambridge, MA, USA) following the manufacturer’s instructions.

### 2.4. Histopathological Evaluation and Immunohistochemistry Assay of the Adipose Tissue

Fresh eWAT, iWAT, and BAT from mice in the NC, HFD, and SZ-A groups were fixed in 4% paraformaldehyde solution, embedded in paraffin wax, and subsequently cut into 5-µm sections for hematoxylin-eosin (H&E) staining. For immunohistochemistry analysis, paraffin-embedded sections were dehydrated, antigen repaired, and incubated overnight at 4 °C with diluted primary antibody. Subsequently, the sections were rinsed in PBS and cultivated with a secondary antibody for 1 h at room temperature. This was followed by color development using ready-to-use diaminobenzidine (DAB) solution. F4/80 and CD86 were used to label macrophage and M1-type macrophage in adipose tissue, respectively. Histological images were taken using a microscope imager (Cytation5, Biotek, Winooski, VT, USA) at 100× and 400× magnification.

### 2.5. RNA Sequencing and Data Analysis

RNA extraction and sequencing analyses were performed by Novogene (Beijing, China). Briefly, isolating the total RNA from eWAT using TRIzol. The total amounts and integrity of RNA were assessed using the RNA Nano 6000 Assay Kit of the Bioanalyzer2100 system (Agilent Technologies, Santa Clara, CA, USA). Then, the extracted mRNA was purified from total RNA using poly-T oligo-attached magnetic bead, followed by fragmentation. The library fragments were purified with the AMPure XP system (Beckman Coulter, Beverly, MA, USA). The PCR product was purified by AMPure XP beads. Finally, the library was obtained. The Agilent Bioanalyzer 2100 system and qRT-PCR were used to assess the library quality. The library was sequenced using the Illumina Novaseq platform, and 150 bp paired-end reads were generated after cluster generation. RNA sequencing data were uploaded to the Gene Expression Omnibus (GEO) under accession number (GSE214618). 

HISAT2 (version 2.0.5) software and paired-end clean reads were used to align the high-quality reads to the Ensemble mouse (mm10/GRCm38) reference genome. Differential expression analysis was performed by the DESeq2 R package (1.20.0), and the resulting *p*-values were adjusted using Benjamini and Hochberg’s approach to control the false discovery rate. Transcripts with a padj < 0.05 and |log2(foldchange)| > 1.5 were set as the threshold for differentially expressed genes (DEGs). Gene Ontology (GO) and Kyoto Encyclopedia of Genes and Genomes (KEGG) function enrichment analyses of DEGs were implemented by Novogene (Beijing, China). Furthermore, an analysis of interactions among the top 100 DEGs (based on experimental and curated databases) and functional enrichment analysis (concerning biological processes, cell components, UniProt keywords, and KEGG or Reactome pathways) was performed according to the STRING database (https://string-db.org; version 11.5, accessed on 31 August 2022).

### 2.6. Western Blot

The epididymal adipose tissue samples were homogenized in ice-cold lysis buffer containing complete tablets, mini EASYpack (Roche, Basel, Switzerland), and 1 mmol/L phenylmethanesulfonyl fluoride (Solarbio, Beijing, China) for 30 min. The homogenate was centrifuged at 4 °C with a speed of 12,000× *g* for 15 min and the supernatant was collected. Protein concentration was determined using the BCA assay (Thermo Fisher Scientific, Waltham, MA, USA). Next, the proteins were separated using 12% SDS-PAGE and transferred to a polyvinylidene difluoride membrane. The membranes were blocked with 5% skimmed milk in tris-buffered saline with Tween 20 (TBST) for 2 h at room temperature, then incubated with the following primary antibodies: β-actin (1:1000), p-ACC (1:1000), ACC (1:1000), p-HSL (1:1000), HSL (1:1000), PPARα (1:1000), and ATGL (1:1000) overnight at 4 °C. Subsequently, the membranes were rinsed using TBST three times and incubated with HRP-conjugated anti-rabbit secondary antibody (1:5000) for 1 h at room temperature. The protein expression bands were detected using enhanced chemiluminescence (Easybio, Beijing, China) and visualized using a Tanon-4600SF chemiluminescence imager (Tanon Science & Technology Co., Ltd., Shanghai, China).

### 2.7. Real-Time Polymerase Chain Reaction Analysis

Total RNA was isolated from eWAT using the TRIzol reagent (Invitrogen, CA, USA) and the RNA was quantified using a Nano-300 Micro-Spectrophotometer (AllSheng, Hangzhou, China). cDNA was synthesized from 1 µg of RNA using a reverse transcription system (Promega, Madison, WI, USA), following the manufacturer’s instructions. Real-time RT-PCR was performed using a protocol consisting of 40 cycles of 30 s at 95 °C, 10 s at 95 °C, and 30 s at 60 °C. Melting curve analysis was used to determine the purity of the PCR products. The 2−∆∆CT method was used to calculate the relative expression of each gene. The expression level of each gene was normalized to that of the internal control-*Ppia*. The used primers in this experiment are listed in Table S1 in the Supplementary Information.

### 2.8. Software and Statistical Analysis

The relative protein expressions, adipocyte area, and positive area were quantified using ImageJ software (NIH, Bethesda, MD, USA). Graphs were generated using Prism 8 software (GraphPad Software Inc., San Diego, CA, USA). Data are expressed as mean ± standard error of the mean (SEM). Multiple groups were compared using one-way or two-way analysis of variance (ANOVA) followed by Tukey’s test, depending on the experiment. The two experimental groups were evaluated using a *t*-test. Statistical significance was defined as *p <* 0.05.

## 3. Results

### 3.1. Protective Effect of SZ-A on HFD-Induced Obese Mice

**Figure 1:**
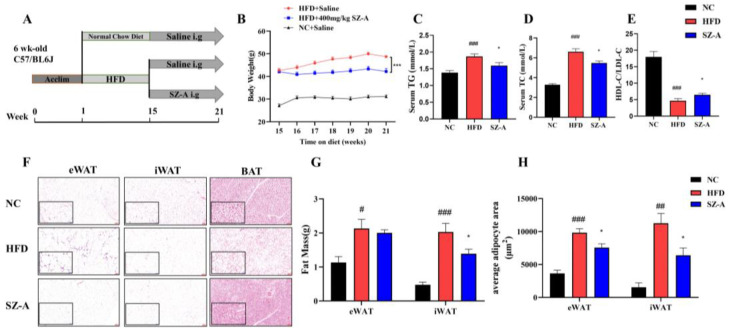
(**A**) Schematic diagram of the experimental procedure (*n* = 10/group). (**B**) SZ-A (400 mg/kg i.g.) significantly inhibited HFD-induced weight gain. (*n =* 10/group) (**C**–**E**) Effects of SZ-A (400 mg/kg) on serum levels of TGs (**C**), TC (**D**) and HDL-C/LDL-C (**E**) (*n* = 6/group) (**F**) Representative images of hematoxylin-eosin (H&E) staining of eWAT, iWAT, and BAT to observe lipid droplet size. (Scale bar, 100 μm in red and 50 μm in blue). (**G**) Total fat mass. (**H**) Quantification of eWAT and iWAT adipocyte area (*n =* 3~5/group). Values represent mean ± SEM. (# *p <* 0.05, ## *p <* 0.01, ### *p <* 0.001 HFD vs. NC and * *p* < 0.05, *** *p* < 0.001 HFD vs. SZ-A).

Our previous research found that SZ-A inhibited body weight gain in HFD mice in a dose-dependent manner and that a dose of 400 mg/kg had the most notable effect on body weight inhibition [11]. In this study, six-week-old C57BL/6J mice were fed an HFD for 14 weeks, and then administered SZ-A (i.g.) for an additional six weeks with continuous HFD feeding (Figure 1A). Consistent with our previous findings, HFD-fed mice showed a significant increase in body weight, whereas mice treated with 400 mg/kg SZ-A showed a significant effect in decreasing body weight (Figure 1B). The effects of SZ-A on lipid metabolism and obese adipose inflammation were studied in detail in mice treated with 400 mg/kg of SZ-A. Compared with the HFD group, the SZ-A-treated mice group demonstrated significantly lower serum levels of triglycerides (TGs) and total cholesterol (TC), and a higher ratio of high-density lipoprotein cholesterol (HDL-C) to low-density lipoprotein cholesterol (LDL-C) (Figure 1C–E). The fat accumulation in the eWAT, iWAT, and BAT was significantly high in the HFD group (Figure 1F). We performed MRI on mice of HFD and SZ-A groups and found the area of subcutaneous fat in the SZ-A group was significantly fewer than that in the HFD group (Figure S1 in the Supplementary Information). Then, we weighed the wet weight of the eWAT and iWAT, and the result showed that SZ-A treatment significantly reduced the mass of iWAT in the SZ-A group in comparison with that in the HFD group (Figure 1G). SZ-A treatment significantly reduced the mass of iWAT in the SZ-A group in comparison with that in the HFD group (Figure 1G). The results of the adipocyte size analysis showed that SZ-A-treated mice exhibited a significant reduction in the volume of enlarged adipocytes in eWAT and iWAT compared to the HFD mice (Figure 1H).

### 3.2. SZ-A Improves Lipid Metabolism

**Figure 2:**
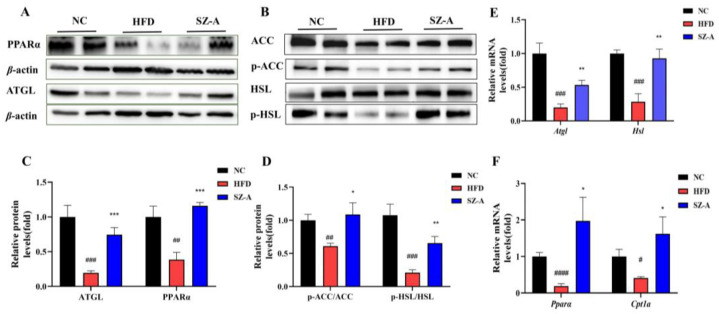
(**A**) Protein expressions of PPARα and ATGL in eWAT were determined using western blotting. (**B**) Protein expressions of ACC, p-ACC, HSL, and p-HSL in eWAT were determined using western blotting (*n* = 5~6/group). (**C**) and (**D**) Protein expression histograms. (**E**) and (**F**) Relative mRNA expression levels of genes, including *Atgl*, *Hsl*, *Pparα*, and *Cpt1a* (*n* = 4~6/group). Values represent mean ± SEM. (# *p <* 0.05, ## *p <* 0.01, ### *p <* 0.001, #### *p* < 0.0001, HFD vs. NC and * *p <* 0.05, ** *p* < 0.01, *** *p* < 0.001 HFD vs. SZ-A).

Western blotting was used to examine the proteins associated with lipid synthesis and lipolysis, and the results are shown in Figure 2A,B. These results suggested that HFD could significantly down-regulate the protein levels of lipolytic enzymes, whereas SZ-A could significantly up-regulate their expression. The expression of peroxisome proliferator-activated receptor-alpha (PPARα), adipose triglyceride lipase (ATGL), phosphorylated hormone-sensitive triglyceride lipase (p-HSL), and phosphorylated acetyl coenzyme A carboxylase (p-ACC) was significantly reduced in the eWAT in HFD groups. However, after SZ-A treatment, PPARα and ATGL expression levels were restored. These results also indicated that SZ-A treatment promoted the phosphorylation of ACC and HSL. Moreover, RT-PCR results (Figure 2E,F) showed that *Pparα*, *Atgl*, *Hsl*, and carnitine palmitoyl transferase 1A (*Cpt1a*) transcription levels were also increased in the SZ-A-treated group. Based on the results, regarding the mechanism of SZ-A reducing obesity in adipose tissue, it can be concluded that SZ-A could promote triglyceride catabolism and oxidation by up-regulating the expression of PPARα, ATGL, and p-HSL and promoting p-ACC to reduce fatty acid synthesis. Therefore, SZ-A may have the potential to improve lipid metabolism in the adipose tissue of obese mice.

### 3.3. The Analysis of Differentially Expressed Genes in the Epididymal Fat 

**Figure 3:**
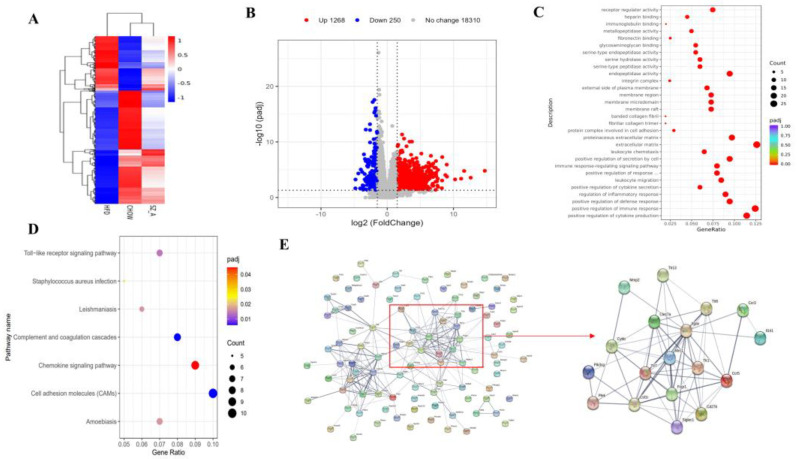
(**A**) Heatmap of NC, HFD, and SZ-A groups differential genes (Padj < 0.05, |log2(foldchange)|> 1.5) in eWAT (**B**) Volcano plot of the differentially expressed genes (Padj < 0.05, |log2(foldchange)| > 1.5) in eWAT of HFD control and SZ-A group (*n* = 7/group). Red and blue represent the high and low expression of genes in the SZ-A group, respectively. (**C**) GO analysis on the down-regulated genes in the SZ-A group. (**D**) KEGG analysis on the down-regulated genes in the SZ-A group. (**E**) Protein-protein interaction network analysis of the top 100 differential genes using String database.

The transcriptional changes induced by SZ-A were also investigated via RNA sequencing on the eWAT of NC, HFD, and SZ-A-treated HFD mice (*n* = 7/group). As shown in Figure 3A, an HFD resulted in abnormal gene expression and SZ-A treatment restored the gene expression. The volcano plot shows that 1518 genes were differentially expressed in the HFD control and SZ-A groups (Padj < 0.05, fold change > 1.5, Figure 3B). Among these, 1268 genes were up-regulated and 250 were down-regulated. To further identify the predominant biological pathways affected by SZ-A, we analyzed the DEGs and performed GO and KEGG enrichment analyses of the down-regulated genes. GO enrichment analysis showed that inflammation-related gene transcription obviously exhibited a decrease in the SZ-A group (Figure 3C). This included genes involved in leukocyte chemotaxis, regulation of cytokine secretion, activation of cells involved in the immune response, and positive regulation of the immune response. KEGG enrichment analysis showed that the DEGs were primarily associated with Toll-like receptor (TLR) signaling pathways, cell adhesion molecules, and chemokine signaling pathways (Figure 3D). In addition, analysis of the top 100 DEGs using the STRING database showed that *Tlr1*, *Tlr7*, *Tlr8*, and *Tlr13* were at network nodes (Figure 3E). These results suggest that SZ-A regulates genes and pathways associated with adipose tissue inflammation in HFD mice, presumably by inhibiting the Toll-like receptor pathways to exert anti-inflammatory effects. 

### 3.4. SZ-A Improves the Inflammatory Response of Epididymal Fat

**Figure 4:**
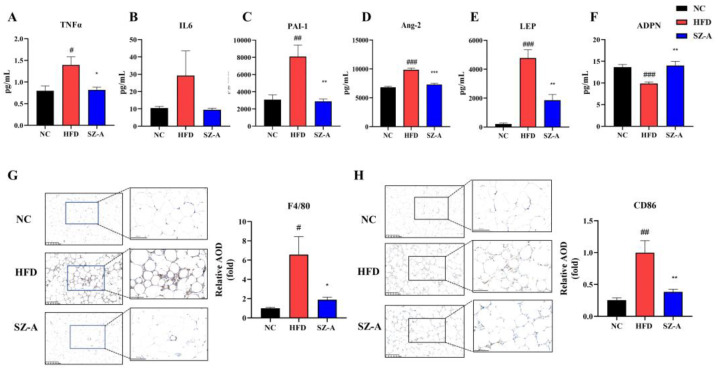
Inflammatory factors TNFα (**A**), IL6 (**B**), PAI-1 (**C**), Ang-2 (**D**), LEP (**E**) and ADPN (**F**) in the serum of the three groups of mice (*n* = 6/group). Representative images of F4/80 (**G**) and CD86 (**H**) immunohistochemical staining of the eWAT (Scale bar, 100 µm, and 200 µm). The histograms indicate quantification of F4/80 or CD86 positive area per field (*n* = 6/group). Values represent mean ± SEM. (# *p <* 0.05, ## *p <* 0.01, ### *p <* 0.001 HFD vs. NC and * *p* < 0.05, ** *p* < 0.01, *** *p <* 0.001 HFD vs. SZ-A).

Transcriptomic data suggest that SZ-A may have an ameliorative effect on adipose tissue inflammation in obese mice. Based on this, we further evaluated inflammatory cell infiltration, inflammatory factor, and gene transcription in the eWAT of mice in NC, HFD, and SZ-A-treated groups using immunohistochemistry, multi-factor assay, and real-time PCR, respectively. The serum multi-factor assay showed that the pro-inflammatory factors expression levels, including TNFα, IL6, PAI-1, Ang-2, and LEP, were significantly increased in the serum of HFD mice. However, SZ-A treatment obviously downregulated the expression of these pro-inflammatory factors (Figure 4A–E). In addition, SZ-A promoted the expression of ADPN in mouse serum (Figure 4F). Therefore, we hypothesized that SZ-A might alleviate the chronic inflammation of adipose tissue induced by HFD. Immunohistochemical results (Figure 4G,H) showed that F4/80 and CD86 positive markers were higher in the eWAT of mice in the HFD group than in the NC group, showing a dendritic structure. This indicated that the HFD increased the infiltration of pro-inflammatory M1 macrophages in eWAT. After treatment with SZ-A, the expression level of CD86 was significantly reduced, suggesting an alleviation effect of SZ-A on epididymal fat inflammation (Figure 4H).

**Figure 5:**
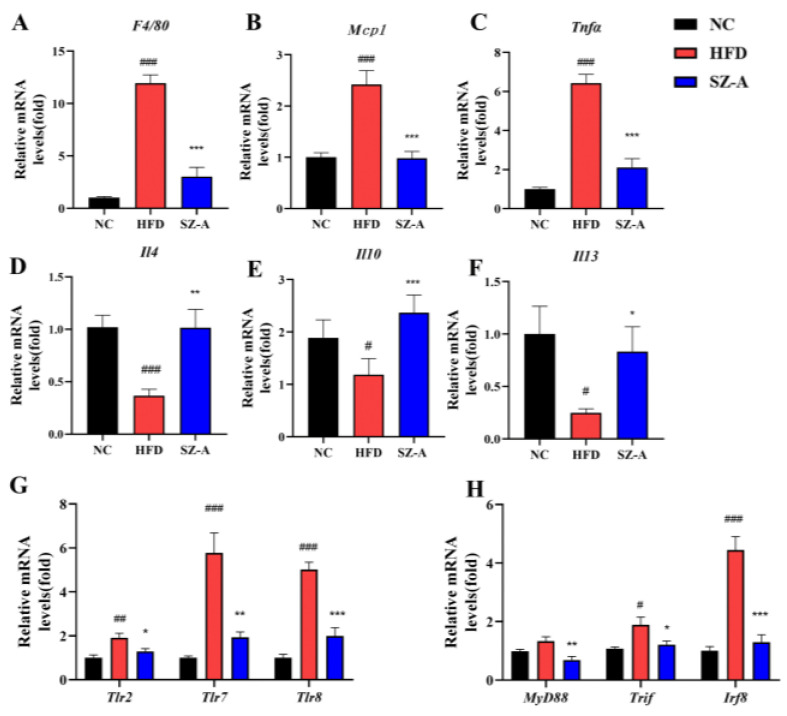
(**A**–**C**) Relative mRNA expression levels of the pro-inflammatory-related factors, including *F4/80*, *Mcp1*, and *Tnfα*, in the three groups. (**D**–**F**) Relative mRNA expression levels of the anti-inflammatory-related factors, including *Il4*, *Il10*, and *Il13*, in the three groups. (**G**,**H**) Relative mRNA expression levels of *Tlrs* and downstream genes, including *Tlr2*, *Tlr7*, *Tlr8*, *MyD88*, *Trif*, and *Irf8*. (*n* = 6/group). Values represent mean ± SEM. (# *p <* 0.05, ## *p <* 0.01, ### *p <* 0.001 HFD vs. NC and * *p <* 0.05, ** *p <* 0.01, *** *p <* 0.001 HFD vs. SZ-A).

Herein, RT-PCR was used to further examine the transcription of genes for the pro-inflammatory-related factors (such as *Tnfα*, *Mcp1*, *F4/80*, *Tlr2*, and so on) and the anti-inflammatory-related factors (including *Il4*, *Il10*, and *Il13*) in the eWAT of the obese mice (Figure 5). The results showed that the transcription of *Tnfα*, *Mcp1*, and *F4/80* was decreased in the eWAT of SZ-A-treated mice compared to those in the HFD group (Figure 5A–C), whereas the transcription of *Il4*, *Il10*, and *Il13* was increased (Figure 5D–F). The mRNA expression levels of *Tlr2*, *Tlr7*, *Tlr8*, and their downstream genes MyD88, TIR-domain-containing adaptor-inducing interferon (*Trif*), and interferon regulatory factor 8 (*Irf8*) were also examined, and the results showed that SZ-A significantly decreased the transcript levels of these genes in the toll-like receptor pathway (Figure 5G,H).

## 4. Discussion

It has been verified that obesity can cause chronic low-grade inflammation and increase the risk of systemic metabolic dysfunction associated with obesity-linked disorders. In addition to being an energy storage organ, the adipose tissue is also a critical endocrine organ, which can release various bioactive substances with pro-inflammatory or anti-inflammatory activities, such as adipose-derived secreted factors and adipokines. SZ-A, an extract of *Morus alba* L., was approved to treat T2DM in 2020. Besides, SZ-A also showed potential in decreasing weight gain in HFD-mice and in vitro anti-inflammatory. In this study, mice were fed with HFD for 14 weeks to create an obesity model. The most effective dose was selected at 400 mg/kg SZ-A, and this dose was used to conduct an in-depth study of the effects of SZ-A on the regulation of lipid metabolism and inflammation of adipose tissue.

Firstly, the adipose tissue of HFD-induced obese mice in pathological sections showed that SZ-A significantly reduced the size of adipocytes in both WAT and BAT. It is well known that central obesity is dangerous to human health [14]. Therefore, in this study, we selected the eWAT (abdominal adipose representative adipose tissue) from obese mice for lipid metabolism-related analysis. Our findings suggest that SZ-A inhibits the catalytic capacity of ACC in epididymal adipose tissue by promoting its phosphorylation, thereby inhibiting the de novo synthesis of fatty acids. As the core enzyme that catalyzes lipolysis in adipocytes, ATGL specifically catalyzes triglyceride catabolism, whereas HSL is primarily responsible for catalyzing diglyceride catabolism. It has been reported that lipolysis is reduced in ATGL-deficient mice, and lipid accumulation occurs in almost all tissues throughout the body [15]. The catalytic activity of HSL is primarily related to its phosphorylation site. Phosphorylation of the Ser563 site has been found to stimulate HSL activity and may enhance its catalytic activity by promoting the translocation of HSL to lipid droplets [16]. In this study, SZ-A significantly promoted the protein expression of ATGL, promoted the phosphorylation of HSL (Ser563), and increased the mRNA levels of both proteins. In addition, SZ-A promoted the protein expression and transcription of *Pparα* as well as the transcriptional level of *Cpt1a*. PPARα is a transcription factor that belongs to the member of the nuclear hormone receptor family and is a fatty-acid sensor. Activation of PPARα increases the catabolic oxidation of fatty acids and facilitates the transport of long-chain fatty acids to mitochondria via CPT1A, which in turn increases the fatty acids’ oxidative catabolism [17]. Accordingly, the results of this study suggest that SZ-A reduces fatty acid synthesis at its source as well as enhances fatty acid catabolism and oxidation by promoting the breakdown of triglycerides and diglycerides in eWAT, thereby reducing lipid accumulation, achieving weight loss, and improving lipid metabolism.

As a critical endocrine organ, the adipose tissue secretes many adipokines and inflammatory factors. Obesity triggers chronic inflammation, which is a sign of adipose tissue dysfunction and metabolic dysregulation. TNF-α and IL6 are two major pro-inflammatory factors in adipose tissue, and they are usually expressed at elevated levels in the serum and adipose tissue of obese individuals and involved in obesity-related systemic insulin resistance [4]. Compared with the non-obese population, PAI1 and Ang-2 levels were obviously higher in the obese population. PAI-1 is a member of the serine protease inhibitor family and plays an important regulatory part in the fibrinolytic process and thrombosis. Although the liver is the primary source of PAI-1, some studies have demonstrated that visceral fat in obese people is the predominant cause of elevated PAI-1 levels. Moreover, the elevated PAI-1 expression triggers cardiovascular diseases, such as atherosclerosis [18]. Ang-2 is mainly secreted by adipose tissue, and while getting obese, its level would be increased. Ang-2 plays a substantial role in the development of obesity-type hypertension and insulin resistance [19]. Clinical data show that serum LEP levels are elevated and ADPN levels are reduced in obese individuals. LEP primarily acts on the central nervous system and regulates energy metabolism by suppressing appetite. Circulating LEP levels are proportionally correlated with fat mass, and obese individuals have higher levels of LEP but no anorexic response, a phenomenon known as leptin resistance [20]. Furthermore, LEP has pro-inflammatory activity, and its elevated levels are associated with insulin resistance and liver fibrosis in obese individuals [21]. To date, ADPN is an adipokine that is negatively associated with obesity, but its levels are higher in healthy people who have gained weight and are lower in a metabolically disturbed state of obesity. Increased ADPN expression is expected to increase insulin sensitivity and reduce cardiovascular risk in obese people [22]. In this study, the results of the serum multi-factor assay showed that the SZ-A-treated group showed noteworthy lower serum levels of TNFα, IL6, PAI1, Ang-2, and LEP and higher serum expression levels of ADPN compared to the HFD group. SZ-A exhibits excellent in ameliorating HFD-induced organismal and adipose tissue inflammation in obese mice.

Obesity usually induces macrophage accumulation and inflammation in adipose tissues. Adipocytes in obese people secrete more pro-inflammatory and chemotactic factors, causing large numbers of macrophages to recruit into adipose tissue and triggering persistent low-grade chronic inflammation. Low-grade chronic inflammation is always affiliated with chronic diseases, such as insulin resistance, NAFLD, and cardiovascular pathology. The development of adipose tissue inflammation is largely dependent on inflammatory cells, such as macrophages recruited to the adipose tissue, particularly pro-inflammatory M1-type macrophages. M1-type macrophages have been reported to be the major generator of pro-inflammatory cytokines and involve dead or dying adipocytes in adipose tissue, modeling a typical coronal structure [23]. The results of the eWAT transcriptome analysis in mice display that SZ-A has a regulatory role in inflammatory genes and pathways in epididymal fat. Therefore, we examined inflammatory factors and cells in the eWAT of HFD-induced obese mice. The results of the immunohistochemical analysis showed that SZ-A treatment could remarkably reduce the counts of both total macrophages and M1-type cells. SZ-A treatment significantly down-regulated the transcription of the pro-inflammatory factors *Tnfα* and *Mcp1*, which may be related to the reduction of the count of macrophages in adipose tissue after SZ-A treatment. This further suggested that SZ-A achieved the amelioration of improving adipose tissue inflammation by reducing macrophage infiltration in adipose tissue, which in turn significantly inhibited the expression of pro-inflammatory factors *Tnfα* and *Mcp1* and up-regulated the gene transcription of anti-inflammatory factors *Il4*, *Il10*, and *Il13.*

Numerous studies have confirmed that Toll-like pattern recognition receptors play an essential role in mediating the proinflammatory effects of free fatty acids [24]. In this study, transcriptome analysis showed that SZ-A could down-regulate the transcription of genes in the Toll-like receptor pathway. TLRs are innate immune pattern recognition receptors expressed in different cells and tissues. Upon binding to ligands, TLRs undergo conformational changes, forming homo- or heterodimers that subsequently recruit bridging proteins, such as MyD88 and TIRF, and initiate intracellular signaling cascades. In adipose tissue, the TLR signaling pathway is important for regulating immune responses [25]. Activation of the TLR2 signaling pathway has been reported to be associated with chronic inflammation, which triggers the development of insulin resistance in adipocytes. TLR2 expression is increased in the muscle and adipose tissue of HFD mice, and TLR2 gene silencing improves insulin resistance in these mice. This implies that TLR2 is an important regulator of the inflammatory and metabolic pathways of HFD-induced obesity [26]. TLR7 and TLR8 are expressed in adipocyte endosomes and lysosomes, respectively. They are up-regulated in the adipose tissue and promote metabolic inflammation in obese individuals [27,28]. In this study, the RT-PCR results of eWAT indicated that SZ-A significantly inhibited the transcription of *Tlr2*, *Tlr7*, and *Tlr8* and their downstream bridging proteins, including *MyD88*, *Tirf*, and *Irf8*. Therefore, SZ-A may have a regulatory effect on the Toll-like receptor pathway, and we speculated that the reduction in TLR signaling may be due to a decrease in adipose tissue infiltrated macrophages.

## 5. Conclusions

In this study, SZ-A (six weeks of treatment) improved lipid metabolism and inhibited weight gain in HFD-induced obese mice by inhibiting fatty acid synthase and increasing lipolytic enzyme expression to inhibit fat accumulation. In addition, SZ-A was seen to have a beneficial effect on obesity-induced chronic inflammation of adipose tissue. SZ-A can reduce the expression of pro-inflammatory factors such as PAI1, Ang-2, TNFα, IL6, and LEP; reduce the recruitment of macrophages, especially M1 macrophages; inhibit the transcription of *Tlr2*, *Tlr7*, *Tlr8*, and their downstream bridging proteins *MyD88*, *Tirf*, and *Irf8*; and up-regulate the expression of anti-inflammatory factors *Il4*, *Il10*, and *Il13* at the level of mRNA.

Thus, SZ-A, a natural anti-diabetic agent, has been shown to be effective in improving disorders of lipid metabolism and adipose tissue inflammation in HFD-induced obese mice.

## Data Availability

RNA sequencing data have been submitted to the Gene Expression Omnibus (GEO) under accession number (GSE214618).

## Supplementary Materials

The following supporting information can be downloaded at: https://www.mdpi.com/article/10.3390/nu14235050/s1, Table S1: Primers for RT-PCR; Figure S1: MRI of mice.

## References

[1] James W.P. (2008). WHO recognition of the global obesity epidemic. Int. J. Obes..

[2] Gregor M.F., Hotamisligil G.S. (2011). Inflammatory mechanisms in obesity. Annu. Rev. Immunol..

[3] Sakers A., De Siqueira M.K., Seale P., Villanueva C.J. (2022). Adipose-tissue plasticity in health and disease. Cell.

[4] Ouchi N., Parker J.L., Lugus J.J., Walsh K. (2011). Adipokines in inflammation and metabolic disease. Nat. Rev. Immunol..

[5] Olefsky J.M., Glass C.K. (2010). Macrophages, inflammation, and insulin resistance. Annu. Rev. Physiol..

[6] Effting P.S., Thirupathi A., Muller A.P., Pereira B.C., Sepa-Kishi D.M., Marqueze L.F.B., Vasconcellos F.T.F., Nesi R.T., Pereira T.C.B., Kist L.W. (2022). Resistance Exercise Training Improves Metabolic and Inflammatory Control in Adipose and Muscle Tissues in Mice Fed a High-Fat Diet. Nutrients.

[7] Pemmari T., Hämäläinen M., Ryyti R., Peltola R., Moilanen E. (2022). Cloudberry (*Rubus chamaemorus* L.) Supplementation Attenuates the Development of Metabolic Inflammation in a High-Fat Diet Mouse Model of Obesity. Nutrients.

[8] Qu L., Liang X., Tian G., Zhang G., Wu Q., Huang X., Cui Y., Liu Y., Shen Z., Xiao C. (2021). Efficacy and Safety of Mulberry Twig Alkaloids Tablet for the Treatment of Type 2 Diabetes: A Multicenter, Randomized, Double-Blind, Double-Dummy, and Parallel Controlled Clinical Trial. Diabetes Care.

[9] Lei L., Huan Y., Liu Q., Li C., Cao H., Ji W., Gao X., Fu Y., Li P., Zhang R. (2022). *Morus alba* L. (Sangzhi) Alkaloids Promote Insulin Secretion, Restore Diabetic beta-Cell Function by Preventing Dedifferentiation and Apoptosis. Front. Pharm..

[10] Liu Q., Liu S., Cao H., Ji W., Li C., Huan Y., Lei L., Fu Y., Gao X., Liu Y. (2021). Ramulus Mori (Sangzhi) Alkaloids (SZ-A) Ameliorate Glucose Metabolism Accompanied by the Modulation of Gut Microbiota and Ileal Inflammatory Damage in Type 2 Diabetic KKAy Mice. Front. Pharmcol..

[11] Chen Y.M., Lian C.F., Sun Q.W., Wang T.T., Liu Y.Y., Ye J., Gao L.L., Yang Y.F., Liu S.N., Shen Z.F. (2022). *Ramulus Mori* (Sangzhi) Alkaloids Alleviate High-Fat Diet-Induced Obesity and Nonalcoholic Fatty Liver Disease in Mice. Antioxidants.

[12] Cao H., Ji W., Liu Q., Li C., Huan Y., Lei L., Fu Y., Gao X., Liu Y., Liu S. (2021). *Morus alba* L. (Sangzhi) alkaloids (SZ-A) exert anti-inflammatory effects via regulation of MAPK signaling in macrophages. J. Ethnopharmacol..

[13] Yang S., Mi J., Liu Z., Wang B., Xia X., Wang R., Liu Y., Li Y. (2017). Pharmacokinetics, Tissue Distribution, and Elimination of Three Active Alkaloids in Rats after Oral Administration of the Effective Fraction of Alkaloids from *Ramulus Mori*, an Innovative Hypoglycemic Agent. Molecules.

[14] Gesta S., Tseng Y.H., Kahn C.R. (2007). Developmental origin of fat: Tracking obesity to its source. Cell.

[15] Haemmerle G., Lass A., Zimmermann R., Gorkiewicz G., Meyer C., Rozman J., Heldmaier G., Maier R., Theussl C., Eder S. (2006). Defective lipolysis and altered energy metabolism in mice lacking adipose triglyceride lipase. Science.

[16] Nielsen T.S., Jessen N., Jorgensen J.O., Moller N., Lund S. (2014). Dissecting adipose tissue lipolysis: Molecular regulation and implications for metabolic disease. J. Mol. Endocrinol..

[17] Aloia A., Mullhaupt D., Chabbert C.D., Eberhart T., Fluckiger-Mangual S., Vukolic A., Eichhoff O., Irmisch A., Alexander L.T., Scibona E. (2019). A Fatty Acid Oxidation-dependent Metabolic Shift Regulates the Adaptation of BRAF-mutated Melanoma to MAPK Inhibitors. Clin. Cancer Res..

[18] Shimomura I., Funahashi T., Takahashi M., Maeda K., Kotani K., Nakamura T., Yamashita S., Miura M., Fukuda Y., Takemura K. (1996). Enhanced expression of PAI-1 in visceral fat: Possible contributor to vascular disease in obesity. Nat. Med..

[19] Tabata M., Kadomatsu T., Fukuhara S., Miyata K., Ito Y., Endo M., Urano T., Zhu H.J., Tsukano H., Tazume H. (2009). Angiopoietin-like protein 2 promotes chronic adipose tissue inflammation and obesity-related systemic insulin resistance. Cell. Metab..

[20] de Git K.C., Adan R.A. (2015). Leptin resistance in diet-induced obesity: The role of hypothalamic inflammation. Obes. Rev..

[21] Petrescu A.D., Grant S., Williams E., An S.Y., Seth N., Shell M., Amundsen T., Tan C., Nadeem Y., Tjahja M. (2022). Leptin Enhances Hepatic Fibrosis and Inflammation in a Mouse Model of Cholestasis. Am. J. Pathol..

[22] Singh P., Sharma P., Sahakyan K.R., Davison D.E., Sert-Kuniyoshi F.H., Romero-Corral A., Swain J.M., Jensen M.D., Lopez-Jimenez F., Kara T. (2016). Differential effects of leptin on adiponectin expression with weight gain versus obesity. Int. J. Obes..

[23] Weisberg S.P., McCann D., Desai M., Rosenbaum M., Leibel R.L., Ferrante A.W. (2003). Obesity is associated with macrophage accumulation in adipose tissue. J. Clin. Investig..

[24] Nguyen M.T., Favelyukis S., Nguyen A.K., Reichart D., Scott P.A., Jenn A., Liu-Bryan R., Glass C.K., Neels J.G., Olefsky J.M. (2007). A subpopulation of macrophages infiltrates hypertrophic adipose tissue and is activated by free fatty acids via Toll-like receptors 2 and 4 and JNK-dependent pathways. J. Biol. Chem..

[25] Kim S.J., Choi Y., Choi Y.H., Park T. (2012). Obesity activates toll-like receptor-mediated proinflammatory signaling cascades in the adipose tissue of mice. J. Nutr. Biochem..

[26] Caricilli A.M., Nascimento P.H., Pauli J.R., Tsukumo D.M., Velloso L.A., Carvalheira J.B., Saad M.J. (2008). Inhibition of toll-like receptor 2 expression improves insulin sensitivity and signaling in muscle and white adipose tissue of mice fed a high-fat diet. J. Endocrinol..

[27] Ahmad R., Kochumon S., Thomas R., Atizado V., Sindhu S. (2016). Increased adipose tissue expression of TLR8 in obese individuals with or without type-2 diabetes: Significance in metabolic inflammation. J. Inflamm..

[28] Sindhu S., Wilson A. (2015). Increased Adipose Tissue Expression of Toll-Like Receptor (TLR)-7 in Obese Individuals: Significance in Metabolic Disease. J. Glycom. Lipidom..

