# Supplementary material for: Ramulus Mori (Sangzhi) Alkaloids Ameliorate Obesity-Linked Adipose Tissue Metabolism and Inflammation in Mice
Source: Nutrients. 2022 Nov 27;14(23):5050. doi: 10.3390/nu14235050 (PMC9739644; doi:10.3390/nu14235050)
Supplement: Supplementary file 1 [file nutrients-14-05050-s001.zip › nutrients-1985128-supplementary.pdf]

SupplementaryInformation

Table S1: PrimersforRT-PCR

Ppia-F	AGCTCTGAGCACTGGAGAGA
Ppia-R	GCCAGGACCTGTATGCTTTA
Atgl-F	GACCTGATGACCACCCTTTCC
Atgl-R	TGCTACCCGTCTGCTCTTTCA
Cpt1a-F	CTCCGCCTGAGCCATGAAG
Cpt1a-R	CACCAGTGATGATGCCATTCT
Pparα-F	AGAGCCCCATCTGTCCTCTC
Pparα-R	ACTGGTAGTCTGCAAAACCAAA
Hsl-F	AAGGACTCACCGCTGACTTCC
Hsl-R	GCCTGTCTCGTTGCGTTTGTA
F4/80-F	ACCACAATACCTACATGCACC
F4/80-R	AAGCAGGCGAGGAAAAGATAG
Mcp-1-F	TTAAAAACCTGGATCGGAACCAA
Mcp-1-R	GCATTAGCTTCAGATTTACGGGT
Tnfα-F	CTTCTGTCTACTGAACTTCGGG
Tnfα-R	CAGGCTTGTCACTCGAATTTTG
Il4-F	GGTCACCCCCAGCTAGT
Il4-R	GCCGATCTCTCTCAAGTGAT
Il10-F	CTTACTGACTGGCATGAGGATCA
Il10-R	GCAGCTCTAGGAGCATGTGG
Il13-F	CCTGGCTCTTGCTTGCCTT
Il13-R	GGTCTTGTGTGATGTTGCTCA
Tlr1-F	GGTTGGTGGTCTCGGGTATTG
Tlr1-R	GGCGATGTCACCCCAAAACA
Tlr7-F	ATGTGGACACGGAAGAGACAA
Tlr7-R	GGTAAGGGTAAGATTGGTGGTG
Tlr8-F	GAAAACATGCCCCCTCAGTCA
Tlr8-R	CGTCACAAGGATAGCTTCTGGAA
Tlr13-F	GATCGACGAAGGTGCCTTTAG
Tlr13-R	TGGACTGCTTCAAGAATACCAGA
MyD88-F	AAGAAAGTGAGTCTCCCCTC
MyD88-R	TCCCATGAAACCTCTAACAC
Trif-F	ATGGATAACCCAGGGCCTT
Trif-R	TTCTGGTCACTGCAGGGGAT
Irf8-F	CGGGGCTGATCTGGGAAAAT
Irf8-R	CACAGCGTAACCTCGTCTTC

We have performed MRI on mice of HFD and SZ-A groups by using a Pharma Scan70/16US small animal MRI (Bruker, Ettlingen, Germany). As shown in the following MRI FigureS1, the area of subcutaneous fat in the SZ-A group was significantly fewer than that in the HFD group.

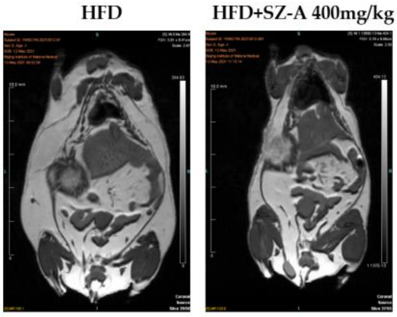

Figure S1. MRI of mice
